# Graph schema and best graph type to compare discrete groups: Bar, line, and pie

**DOI:** 10.3389/fpsyg.2022.991420

**Published:** 2022-12-19

**Authors:** Fang Zhao, Robert Gaschler

**Affiliations:** ^1^Center of Advanced Technology for Assisted Learning and Predictive Analytics, FernUniversität in Hagen, Hagen, Germany; ^2^Department of Psychology, FernUniversität in Hagen, Hagen, Germany

**Keywords:** graph comprehension, group comparison, graph schema, mixing-costs paradigm, hierarchical structure

## Abstract

Different graph types may differ in their suitability to support group comparisons, due to the underlying graph schemas. This study examined whether graph schemas are based on perceptual features (i.e., each graph type, e.g., bar or line graph, has its own graph schema) or common invariant structures (i.e., graph types share common schemas). Furthermore, it was of interest which graph type (bar, line, or pie) is optimal for comparing discrete groups. A switching paradigm was used in three experiments. Two graph types were examined at a time (Experiment 1: bar vs. line, Experiment 2: bar vs. pie, Experiment 3: line vs. pie). On each trial, participants received a data graph presenting the data from three groups and were to determine the numerical difference of group A and group B displayed in the graph. We scrutinized whether switching the type of graph from one trial to the next prolonged RTs. The slowing of RTs in switch trials in comparison to trials with only one graph type can indicate to what extent the graph schemas differ. As switch costs were observed in all pairings of graph types, none of the different pairs of graph types tested seems to fully share a common schema. Interestingly, there was tentative evidence for differences in switch costs among different pairings of graph types. Smaller switch costs in Experiment 1 suggested that the graph schemas of bar and line graphs overlap more strongly than those of bar graphs and pie graphs or line graphs and pie graphs. This implies that results were not in line with completely distinct schemas for different graph types either. Taken together, the pattern of results is consistent with a hierarchical view according to which a graph schema consists of parts shared for different graphs and parts that are specific for each graph type. Apart from investigating graph schemas, the study provided evidence for performance differences among graph types. We found that bar graphs yielded the fastest group comparisons compared to line graphs and pie graphs, suggesting that they are the most suitable when used to compare discrete groups.

## 1. Introduction

Graphs are widely and increasingly employed to visualize quantitative information in science, marketing, sports, politics, etc. (Zacks et al., 2002; Shah et al., 2005; Ratwani et al., 2008; Garcia-Retamero and Cokely, 2017; Padilla et al., 2018, for reviews; Franconeri et al., 2021). Experimental evidence has shown that it is easier to understand, communicate and reason about information when it is presented in graphic representations (Tufte, 1983; Wainer, 1992; Kastellec and Leoni, 2007). While there is a multitude of graph types (Garcia-Retamero and Cokely, 2017; Padilla et al., 2018; Franconeri et al., 2021), some formats seem to be characterized by high typicality (cf. Reimann et al., 2022) and have been in use since the early days of statistical graphing: The pie chart appears in William Playfair's Statistical Breviary of 1801. Line and bar graphs were published by Playfair in 1786 (Spence, 2006). The utility of line and bar graphs has been wellsupported by a body of experimental research (Spence, 2006) and the formats have been integrated into the education curriculums used throughout the world to develop children's ability to read and construct data visualizations (Börner et al., 2019; Franconeri et al., 2021).

One of the most essential questions is how we decode information from graphs. Different cognitive models of graph comprehension (e.g., Pinker, 1990; Lohse, 1993; Padilla et al., 2018) suggest that people have graph schemas stored in long-term memory and that comprehension of a given graph requires that the visually encoded stimulus is matched to the appropriate graph schema (Kosslyn, 1989). For instance, bar graphs and line graphs are characterized by an “L-shaped” graph schema with horizontal and vertical axes that define a Cartesian coordinate system. In contrast, pie charts and doughnut graphs belong to an “O-shaped” graph schema characterized by a circular space defined by polar coordinates (angle and distance from center). While researchers agree that activating the graph schema is the most crucial stage in graph comprehension (Bertin, 1983; Pinker, 1990), it is disputable to what extent different graph types overlap in or share graph schemas. According to one perspective, graph schemas might built on distinct perceptual features (i.e., each graph has its own specific graph schema, Lohse, 1993). Alternatively, one graph schema might be used for different graph types (cf. Ratwani and Trafton, 2008) as it captures the common invariant structure shared by the graph types.

The study by Ratwani and Trafton (2008) examined hypotheses of graph schemas by utilizing a fact-retrieval task (e.g., how many widgets are there in Tray B). Yet group comparisons (e.g., what is the numerical difference of group A and group B) would be a task that would benefit greatly from the power of graphs to enable relational information processing (Zhao and Gaschler, 2021). While there have been earlier studies on bar, line, and pie graphs (Eells, 1926; Simkin and Hastie, 1987; Hollands and Spence, 1992; Shah et al., 1999; Zacks and Tversky, 1999), we aimed to investigate whether or not they share a common graph schema when the task is to assess numerical differences by using the technique suggested by Ratwani and Trafton (2008). Apart from targeting the mental representations used in graph processing, we tested which graph type (bar, line, or pie) is best suited for discrete comparisons.

### 1.1. Graph schema: Perceptual features vs. common invariant structures

By considering the perceptual process as well as short-term memory and long-term memory processes, many theories of graph comprehension have been developed explaining how we extract information from graphs (Pinker, 1990; Lohse, 1993; Shah and Carpenter, 1995; Peebles and Cheng, 2002, 2003; Shah and Hoeffner, 2002; Ratwani and Trafton, 2008). First, pattern recognition methods are used to decipher the visual information in the graph (e.g., length, width, darkness, shape, and position, cf. Bertin, 1983). Second, abstract concepts of the visual information are mentally constructed in the capacity-limited working memory (c.f. Cowan, 2016). Third, conceptual relations are retrieved from long-term memory, and interpretative processes are initiated by activating the graph schema (i.e., a generic scaffold to insert new information into a complex knowledge representation, Simkin and Hastie, 1987). Fourth, the desired information is located and provided if it is contained in the activated mental representation. Otherwise, interrogation processes and inferential processes are prompted to add entries or adapt existing entries to the conceptual message (Pinker, 1990).

Two main assumptions are proposed regarding the structure of graph schemas. The assumption that graph schemas are defined based on specific *perceptual features* suggests that each type of graph is determined by a unique graph schema, which results in different task sets (i.e., active mental configurations needed for processing) for different graphs (Lohse, 1993). This view is in line with the scene perception literature (Potter, 1993, 2012), which suggests perceptual characteristics can trigger specific graph schemas (e.g., objects in a graph, Kosslyn, 1989). Bar graphs would thus activate a unique graph schema and so would line graphs and pie charts. In contrast, the assumption that graph schemas are based on an *invariant structure* suggests that types of graphs are based on certain broad categories or shared common characteristics (Peebles and Cheng, 2002, 2003; Ratwani and Trafton, 2008; Zhao and Gaschler, 2021). Graph schemas are organized hierarchically, combining a general schema and a graph-specific schema (Pinker, 1990). The general schema includes common features of many graphs, such as a Cartesian coordinate system. The graph-specific schemas include the unique features of individual graphs, such as bars with different heights or widths.

### 1.2. Features of bar, line, pie

**Figure 1:**
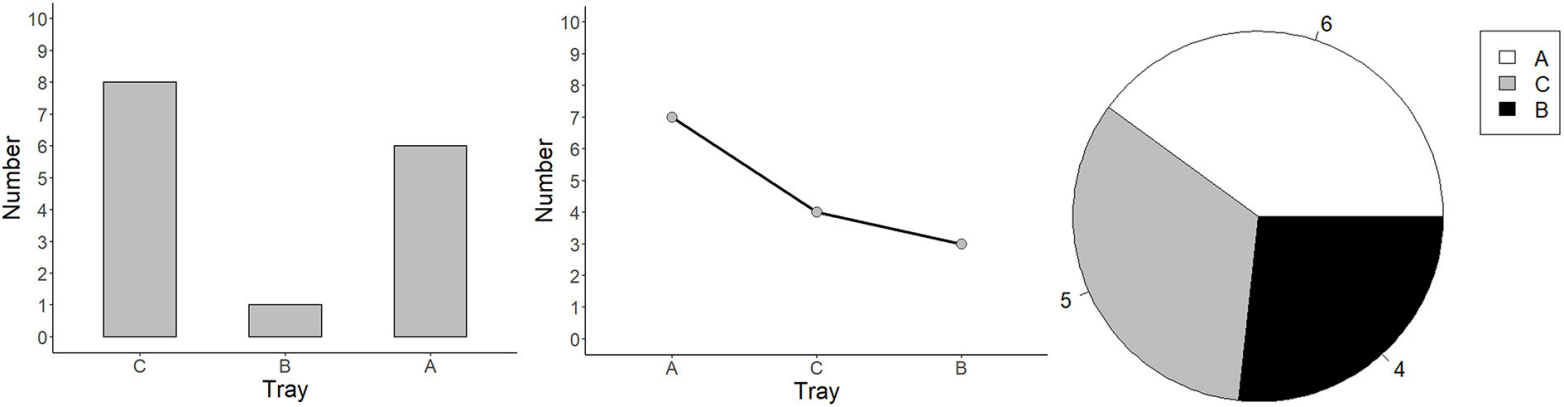
Graphs from Experiments 1–3: bar, line, and pie. Each graph presents the data of three groups: A, B, and C.

Bar graphs consist of bars or closed containers in an L-shaped Cartesian coordinate system with x- and y-axes (Tversky et al., 2000). Bar graphs (Pinker, 1990) pair nominal groups using separate bars on one axis (e.g., group in Figure 1) with ordinally scaled values, indicated by the height of the bars (e.g., number in the group) on the other axis. As bar graphs specify individual entities by separating one group from another, they are ideal for discrete group comparisons (Cleveland, 1984; Cleveland and McGill, 1985; Carswell and Wickens, 1987; Shah et al., 1999; Ward et al., 2015). Viewers are more likely to spontaneously make discrete comparisons in bar graphs, such as “a male's height is higher than that of a female's” (Simkin and Hastie, 1987; Zacks and Tversky, 1999). Bar graphs are less biased than line graphs when describing the relationships of multivariate data (Shah and Shellhammer, 1999). Yet they are not ideal for all tasks. When viewing the means of all groups, bar graphs appear to lead to an underestimation of the grand mean (while dot plots do not; Godau et al., 2016). It is more likely that any particular data point within the bar will be assumed to lie within the distribution (within-the-bar bias, Newman and Scholl, 2012). Also, the length of a vertical bar is often estimated to be 10% longer than the equivalent horizontal bar (Cai et al., 2017).

Line graphs consist of discrete data points that are connected by continuous lines within an L-shaped Cartesian coordinate system (Pinker, 1990). As discrete data points are connected by lines, data points are chunked based on the Gestalt principle of continuity (Todorovic, 2008). Viewers are more likely to spontaneously describe the data in a line graph as a trend (Carswell et al., 1993; Zacks and Tversky, 1999). Lines have advantages when judging change, such as whether the slope of one variable over a range of the other variable is increasing or decreasing (Schutz, 1961; Hollands and Spence, 1992). Viewers tend to integrate information from discrete data points as a single object rather than interpret the data point by point (Carswell and Wickens, 1990; Shah et al., 1999). Thus, discrete comparisons can be difficult in line graphs, as viewers have to trigger a top-down encoding process to focus on single data points along the line (Pinker, 1990). As line graphs can form various shapes (e.g., straight, V-shaped, smooth, curved, scalloped, steep), they are preferred when illustrating data with cause-and-effect relations, quantitative trends, and interactions among variables (Simcox, 1984; Pinker, 1990; Shah and Freedman, 2009; Ali and Peebles, 2013).

Pie charts consist of a circle divided by several lines radiating from a central point in a polar coordinate system (Gillan and Callahan, 2000). It uses angles, slices or arcs (radial areas) and darkness to represent groups, and labels to represent values of groups (see Figure 1). Viewers are more likely to spontaneously make proportion judgments of the division to the whole (Simkin and Hastie, 1987). Thus, pie charts are commonly used to illustrate proportional information in opinion polls (Cleveland and McGill, 1985; Spence and Lewandowsky, 1991) and they are utilized for teaching children fractions, for instance, that 1/3 is greater than 1/4 (Wainer, 1992). Moreover, viewers often estimate proportions by referencing to an anchor (e.g., a quarter, half and three quarters, Eells, 1926). The reaction time (RT) and the error rate of proportion judgments increase as the difference between the segment's size and the anchor increases (Gillan and Callahan, 2000). Pie charts are superior to bar graphs for comparisons involving combinations of components, such as comparing A + B vs. C + D (Spence and Lewandowsky, 1991). Most viewers read pie charts clockwise and the accuracy of comparisons can be supported by ordering the segments by size (Huestegge and Pötzsch, 2018). Pie slices, however, cannot be easily compared, as all radii of pie slices are equal in their radial positions and, to be compared, the pie slices should be mentally spread out horizontally to compare their length (Secrist, 1920, S. 166). Additionally, when labels are positioned within the slices of a pie chart, it can be difficult to extract the referents due to alignment issues, and when labels are displayed outside the pie (in a circular order) they can increase clutter (Huestegge and Pötzsch, 2018).

### 1.3. Switching between graph types

Adopting experimental techniques from basic research on action regulation seems promising when investigating how data graphs are processed. Borrowing from task switching (Rogers and Monsell, 1995; see for a review, Kiesel et al., 2010), Ratwani and Trafton (2008) used mixing costs (Los, 1996) to track which data graphs are processed based on same vs. different schemas in a task. They reasoned that only one graph schema can be active at a time. If the graph presented in a current trial does not fit to the schema still activated from the last trial, deactivating the previous schema and activating the appropriate one will consume time. In blocks with one type of graph, one schema can remain active throughout, so no extra time would be needed to unload and load each schema. The same should be true in blocks with two different types of graphs if these types are processed using the same schema. Ratwani and Trafton (2008) interpreted the slower RT in mixed blocks compared to pure blocks as an indicator suggesting that the two graph types are based on different schemas. A previous study (Zhao and Gaschler, 2021) adopted the mixing-costs paradigm to examine the graph schemas of bar graphs, dot plots, and tally charts. Processing time was similar (no mixing costs) in pure vs. switch vs. non-switch conditions when bar graphs were paired with dot plots. However, processing time was different (mixing costs) in pure vs. switch vs. non-switch conditions when tally charts were mixed with bar graphs or dot plots. This suggested that bar graphs and dot plots are built on the same schema, yet tally charts are built on a schema distinct from that of bar graphs and dot plots.

**Figure 2:**
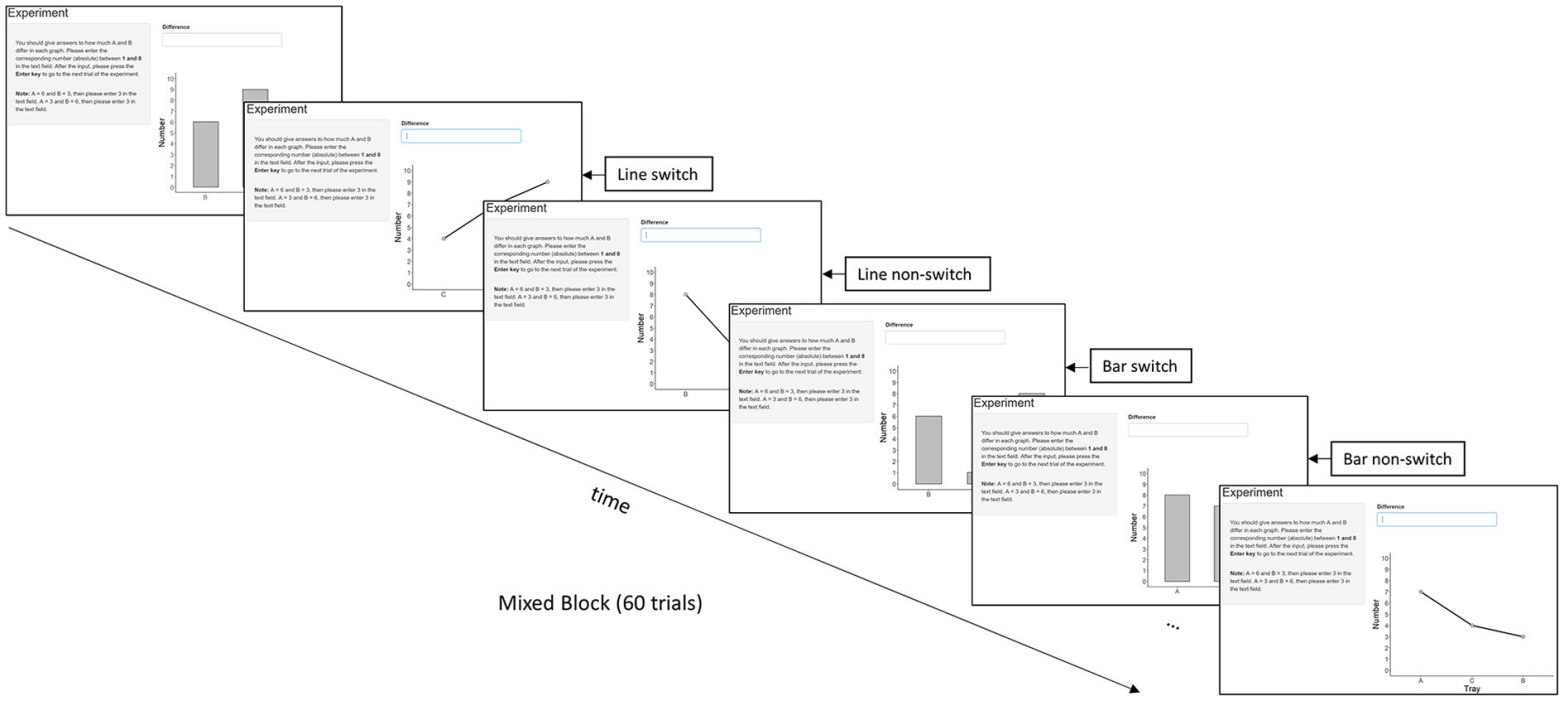
Conditions in a mixed block with switch and non-switch conditions taking Experiment 1 (bar graphs vs. line graphs) as an example. Experiment 2 compared bar graphs and pie graphs, and Experiment 3 compared line graphs and pie graphs.

However, mixing costs might not be the best measure to make inferences about graph schemas. Longer RTs in mixed blocks compared to pure blocks might not be exclusively attributed to different graph schemas being involved. One can, for instance, speculate that effort, motivation or fatigue might differ in pure vs. mixed blocks. Hence, it seems promising to use a measure below the block level that can also be related to a potential deactivation and activation of graph schemas. In the task-switching literature, switch costs are regularly used to assess the need to deactivate and activate task representations (Rogers and Monsell, 1995; Kiesel et al., 2010). Longer RTs in trials with a task switch compared with task repetition are assessed within mixed blocks. Here we apply switch costs to trials with different data graph types, aiming to learn about graph schemas. Taking Experiment 1 (bar vs. line; Figure 2) of the current study as an example, different trial types are characterized by their transition. *Bar switch* refers to a line graph preceded by a bar graph; *bar non-switch* refers to a bar graph preceded by a bar graph; *line switch* refers to a bar graph preceded by a line graph; *line non-switch* refers to a line graph preceded by a line graph. If graphs in a pair are built on distinct schemas, it should take longer to mentally configure the appropriate schema than if they are processed using the same schema, resulting in time costs when switching from one graph type to the next. Conversely, if different graph types are processed using the same schema, RTs in switch trials can be as short as in no-switch trials.

This study adopted the switching paradigm, as it can more appropriately attribute delays in RTs to differences in graph schemas. Each experiment had three trial types: pure vs. switch vs. non-switch. We focused only on comparisons between switch and non-switch trials. Each of our three experiments targeted a pair of graph types, for example, bar vs. line in Experiment 1. Using two graph types ensures that switch trials and non-switch trials are equally frequent. A discrete comparison task (cf. Zhao and Gaschler, 2021) was used based on the following cognitive processes (Kosslyn, 1980; Ullman, 1984; Simkin and Hastie, 1987; Pinker, 1990; Gillan and Callahan, 2000): The first process is *scanning* positions and distances of the compared groups in a graph. The second is *projecting* horizontal rays from the height of the graph to the axis from where they extract the values of the relevant groups. When projection is inadequate, superimposition is activated (Spence and Lewandowsky, 1991) by rotating one group until it aligns with the other. It is important to note that projection can be conducted *via* a routine, where higher bars are attended first (cf. Michal and Franconeri, 2017). The third process is *comparing* two components by adjusting the unaligned border of one component to the other and by applying arithmetic operations and logical inference rules. In a graph, the positions of the groups can also influence the time needed for the comparison. For instance, a previous study showed that the adjacent groups were compared faster than the non-adjacent groups (Zhao and Gaschler, 2021). Thus, we also considered the position difference in the current study.

### 1.4. Research questions

According to the assumption that graph schemas are based on perceptual features, all pairings of graph types should lead to switch costs as they all differ in crucial features. According to the assumption that graph schemas are defined by a common invariant structure, only some pairings will lead to switch costs. Bar graphs and line graphs use heights, points and lines to display quantitative information in a Cartesian system (thus, they share crucial features), whereas pie charts use angular slices in a polar coordinate system (thus, they do not share crucial features with bar and line graphs). Therefore, if the underlying graph schemas share a common invariant structure, switching between pie charts and either bar graphs or line graphs would lead to switch costs while switching between bar graphs and line graphs would not (or to a much lesser extent).

Ratwani and Trafton (2008) used a fact-retrieval task to examine the features of graph schemas. However, it is not yet clear whether the results can be replicated by using discrete comparisons, as line graphs and pie charts can be disadvantageous for discrete comparisons (Secrist, 1920; Pinker, 1990). This leads to the following question and hypotheses.

**Question 1**: Are graph schemas defined by distinct perceptual features for each graph type or by common invariant structures shared between certain graph types?

***Hypothesis 1a***: Bar graphs and line graphs share the same graph schema, which will lead to similar processing times in switch- and non-switch trials.

***Hypothesis 1b***: Pie charts do not share the same graph schema with bar graphs and line graphs, which will lead to longer processing times in switch trials compared with non-switch trials.

Bar graphs have been shown to be processed quicker than line graphs and pie graphs for value identification (Ratwani and Trafton, 2008). However, the cognitive processes when identifying values are different from when comparing values (Follettie, 1986; Simkin and Hastie, 1987; Michal and Franconeri, 2017). Moreover, it is not yet clear whether bar graphs are still superior over line graphs and pie graphs when making discrete comparisons with randomized group positions. This leads to the following question and hypotheses.

**Question 2**: Which graph type (bar, line, or pie) is the most suitable for the comparison of groups when it is required to report the exact numerical difference?

***Hypothesis 2a***: Bar graphs will be processed faster than line graphs and pie graphs.

***Hypothesis 2b***: Adjacent groups (in all graph types) will be compared faster than non-adjacent groups.

## 2. General method

### 2.1. Design and materials

Three graph types were compared in three experiments: Experiment 1 compared bar vs. line graphs, Experiment 2 compared bar vs. pie graphs, Experiment 3 compared line vs. pie graphs. All experiments followed a within-subjects design with three experimental blocks (i.e., two pure blocks, one for each graph type, and one mixed block) consisting of 60 trials each and 180 in total. Each trial contained the instruction to compare the groups A and B, a graph, and a text field to type in the answer (see Figure 2). The graphs presented on the screen all depicted the quantities of three groups (A, B, and C), ranging from 1 to 9. The participants compared the quantities of two of the groups (A and B). The values always varied, which led to group differences with values between 1 and 8. The position of the three groups (A, B, and C) was randomized from 1 to 3. Therefore, the position difference of group A and group B could be either 1 (next to each other), or 2 (group A and group B separated by group C). Participants were allowed to take a break after each block. The experiment was programmed in R by using the package *Shiny*, and each graph was randomly generated.

Based on the switching paradigm, we only compared RTs (i.e., onsets from displaying the graphs to pressing the Enter key) in switch and non-switch trials in the mixed block (e.g., one block of both bar graphs and line graphs, see Figure 2). A repeated-measures analysis of variance (ANOVA) was separately performed in each experiment with the following factors: trial type (switch vs. non-switch), graph type (e.g., bar vs. line), and position difference (of A and B: 1 vs. 2, for adjacent vs. non-adjacent position). The raw data are available online (Zhao, 2022). Due to characteristics of our design, an analysis of mixing costs (instead of switch costs) with regard to our research questions was not feasible (see below, Footnote 1 for details).

### 2.2. Procedure

Participants were tested in a quiet room and informed about the aim of the study. They answered ten questions on a 6-point Likert scale regarding their subjective graph literacy (Garcia-Retamero et al., 2016). For instance, “How well can you work with bar graphs?” (1 = *not well at all* to 6 = *extremely well)*. Afterwards, the experiment was initiated in a browser on a Lenovo Thinkpad T530 laptop with a 12.5-inch display. Participants typed in their demographic data, how frequently they use computers, and agreed with the declaration of consent. They were instructed to report the group difference (e.g., between A and B) in terms of the absolute difference value as accurately and quickly as possible. The participants used the number keys on the keyboard to give their answers and pressed Enter to go to the next trial. All number keys and the Enter key were marked by stickers. The 30-min experiment was part of 8 Bachelor of Science theses and participants received no extra reward.

## 3. Experiment 1: Bar vs. line

### 3.1. Participants

An a priori power analysis using G^*^Power 3.1 (Faul et al., 2009) for a repeated-measures ANOVA testing the main effect of trial type (switch vs. non-switch) while using two graph types (bar vs. line) suggested that a sample size of 40 would allow the detection of an effect of $\eta_{\text{p}}^{2}$ = 0.08 (the effect size of $\eta_{\text{p}}^{2}$ in Zhao and Gaschler, 2021, Experiment 1 was 0.10) at α = 0.05 with a statistical power (1 - β) = 0.95.

Sixty participants (29 females) participated in Experiment 1 (36.0 ± 9.8 years, computer ability with 1 = *never used a computer* to 6 = *everyday use*: 4.5 ± 1.2). The mean age of participants was higher than in many laboratory studies in cognitive psychology as students of FernUniversität in Hagen (state-run distance teaching university in Germany) are older and more heterogeneous in age than students at other universities. On average, graph literacy (Garcia-Retamero et al., 2016) was 4.2 ± 0.8 (1 = *not good at all* to 6 = *extremely good*). All participants had normal or corrected-to-normal vision acuity.

### 3.2. Results

**Figure 3:**
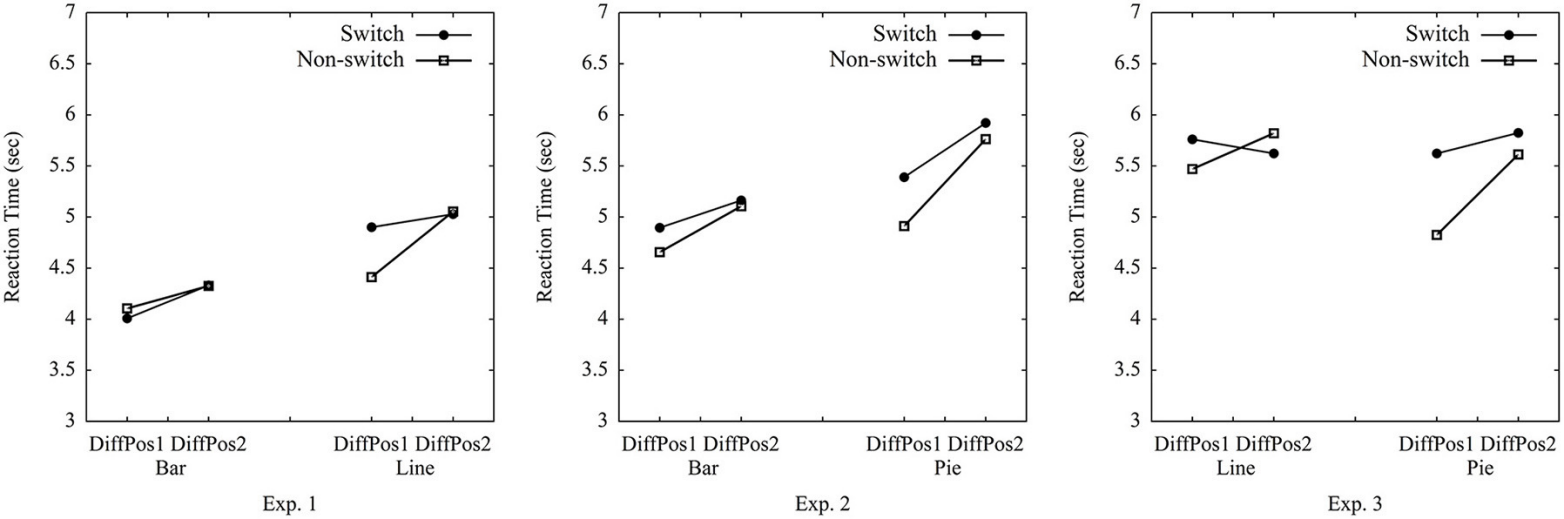
Average median reaction times in switch vs. non-switch trials in Experiments 1–3.

The current study used the median of RTs (cf. Simkin and Hastie, 1987), as the data were left-skewed with a large *SD* (7.651 s). The mean RT was 5.476 s. The average median RT for all trials among all participants was 4.651 s (*SD* = 1.303 s). We conducted the repeated-measures ANOVA on median RTs per participant per condition, while using only two levels of trial type (switch vs. non-switch)^1^ × 2 (graph type: bar vs. line) × 2 (position difference of A and B: 1 vs. 2) (see Table 1, Figure 3). Importantly, the main effect of trial type was marginally significant, [*F*_(1, 59)_] = 3.63, *p* = 0.062, $\eta_{\text{p}}^{2}$ = 0.06 (the estimated Bayes factor was H_01_ = 1.74). There was an interaction of trial type × graph type, [*F*_(1, 59)_] = 6.77, *p* = 0.01, $\eta_{\text{p}}^{2}$ = 0.10. The follow-up test yielded that switch trials were processed with significantly longer RTs than non-switch trials for line graphs, *t*_(59)_ = 5.06, *p* < 0.001, but this was not the case for bar graphs, *t*_(59)_ = −1.08, *p* = 0.29. This suggested that switching between bar graphs and line graphs resulted in switch costs for line graphs, but not for bar graphs (rejecting ***Hypothesis 1a***). A significant main effect of graph type, [*F*_(1, 59)_] = 136.40, *p* < 0.001, $\eta_{\text{p}}^{2}$ = 0.70, indicated that bar graphs were processed quicker than line graphs (confirmed ***Hypothesis 2a***). The main effect of position difference was significant, [*F*_(1, 59)_] = 29.14, *p* < 0.001, $\eta_{\text{p}}^{2}$ = 0.33. So were the interactions of trial type × position, [*F*_(1, 59)_] = 5.74, *p* = 0.02, $\eta_{\text{p}}^{2}$ = 0.09 and trial type × graph type × position, [*F*_(1, 59)_] = 6.20, *p* = 0.02, $\eta_{\text{p}}^{2}$ = 0.10, indicating adjacent groups were processed quicker than non-adjacent groups (confirmed ***Hypothesis 2b***), and the effect of position was larger for non-switch trials in line graphs than in other conditions. No other effect was found, graph type × position, [*F*_(1, 59)_] = 1.11, *p* = 0.30, $\eta_{\text{p}}^{2}$ = 0.02. The analysis of error rates only showed a main effect of graph type, [*F*_(1, 59)_] = 25.49, *p* < 0.001, $\eta_{\text{p}}^{2}$ = 0.30, indicating that bar graphs had significantly lower error rates than line graphs (more details see Supplementary material).

**Table 1:** Average median reaction time (in seconds) between difference of positions of Group A and Group B (DiffPos) in pure trials and switch and non-switch trials for Experiments 1–3.

	**Pure block**		**Mixed block**			
			**Switch**		**Non-switch**	
**Graph type**	**DiffPos1**	**DiffPos2**	**DiffPos1**	**DiffPos2**	**DiffPos1**	**DiffPos2**
**Exp. 1 (*****N*** = **60)**						
Bar *M (SD)*	4.113 (1.169)	4.322 (1.133)	4.008 (0.948)	4.331 (1.367)	4.105 (1.064)	4.326 (1.379)
Line	5.440 (2.090)	5.742 (2.130)	4.900 (1.419)	5.028 (1.527)	4.412 (1.204)	5.053 (1.408)
**Exp. 2 (*****N*** = **41)**						
Bar	5.248 (1.808)	5.258 (1.778)	4.895 (1.417)	5.163 (1.922)	4.656 (1.359)	5.105 (2.322)
Pie	5.924 (2.058)	5.971 (1.764)	5.390 (1.531)	5.921 (2.061)	4.911 (1.322)	5.762 (1.975)
**Exp. 3 (*****N*** = **58)**						
Line	5.671 (1.926)	6.127 (2.211)	5.760 (3.156)	5.622 (2.221)	5.470 (2.847)	5.820 (3.241)
Pie	5.436 (2.478)	5.859 (2.435)	5.622 (2.099)	5.823 (2.557)	4.824 (1.431)	5.612 (2.018)

### 3.3. Discussion

Experiment 1 showed switch costs for line graphs, when bar graphs were paired with line graphs. Yet participants were similarly fast when working on a bar graph following a line graph or another bar graph. These results suggest that bar graphs and line graphs are not processed by the same graph schema, which is inconsistent with previous findings (e.g., Ratwani and Trafton, 2008). Moreover, bar graphs needed less comparison time than line graphs (cf. Zacks and Tversky, 1999). It might be more difficult to locate the height of line graph data points on the y-axis as the slope of the line graph might interfere with the horizontal shift of attention (Todorovic, 2008). Therefore, it is easier to read a bar's height than a data point's value in lines, as values are individually displayed in bar graphs but not in line graphs. In line with previous results, less time was needed when comparing adjacent groups rather than non-adjacent groups (cf. Zhao and Gaschler, 2021).

## 4. Experiment 2: Bar vs. pie

Experiment 2 compared bar graphs and pie graphs. Please note that the position difference (i.e., group assignments) in pie graphs were shown in the legend box (see Figure 1). Graphs were programmed so that the first group was always in white, the second group was in gray, and the third group was in black.

### 4.1. Participants

Forty-one participants (20 females, 29.5 ± 10.7 years, computer ability was 3.8 ± 2.3) took part in Experiment 2. The graph literacy was on average 4.2 ± 0.7.

### 4.2. Results

RTs were left-skewed with a large *SD* (15.264 s). The mean RT was 6.817 s. The average median RT for each trial among all participants was 5.326 s (*SD* = 1.442 s). RT per condition is reported in Table 1, Figure 3. The 2 (trial type: switch vs. non-switch) × 2 (graph type: bar vs. pie) × 2 (position difference of A and B: 1 vs. 2) ANOVA on median RTs per participant per condition showed a main effect of trial type, [*F*_(1, 59)_] = 6.18, *p* = 0.017, $\eta_{\text{p}}^{2}$ = 0.13, suggesting non-switch trials were processed quicker than switch trials (confirmed ***Hypothesis 1b***). Furthermore, there was a main effect of graph type, [*F*_(1, 59)_] = 13.82, *p* < 0.001, $\eta_{\text{p}}^{2}$ = 0.26, indicating bar graphs were processed quicker than pie graphs (confirmed ***Hypothesis 2a***). The main effect of position difference, [*F*_(1, 59)_] = 19.39, *p* < 0.001, $\eta_{\text{p}}^{2}$ = 0.33, suggested that adjacent groups were processed quicker than non-adjacent groups (confirmed ***Hypothesis 2b***). No other effect was found, trial type × position, [*F*_(1, 59)_] = 1.15, *p* = 0.29, $\eta_{\text{p}}^{2}$ = 0.03, graph type × position, [*F*_(1, 59)_] = 2.57, *p* = 0.12, $\eta_{\text{p}}^{2}$ = 0.06, trial type × graph type, and trial type × graph type × position, *Fs* < 1. The analysis of error rates only showed a main effect of position, [*F*_(1, 59)_] = 5.05, *p* = 0.03, $\eta_{\text{p}}^{2}$ = 0.11, indicating that a larger position difference led to lower error rates (more details see Supplementary material).

### 4.3. Discussion

Experiment 2 showed switch costs in terms of differences between switch and non-switch trials, when bar graphs were paired with pie graphs. Comparing groups was quicker when using bar graphs rather than pie graphs, which is consistent with previous studies (Secrist, 1920; Simkin and Hastie, 1987; Huestegge and Pötzsch, 2018). This might be due to disadvantages of a graph schema for pie charts in discrete comparisons. Areas of pie slices involve curved and straight lines and one has to mentally rotate their angles to compare their differences (Gillan and Callahan, 2000; Huestegge and Pötzsch, 2018). Additionally, adjacent groups were compared faster than non-adjacent groups.

## 5. Experiment 3: Line vs. Pie

### 5.1. Participants

Fifty-eight participants (31 females, 40.6 ± 12.7 years, computer ability was 4.2 ± 1.4) took part in Experiment 3. Data on graph literacy was missing for twenty participants, the rest were on average at a level of 4.1 ± 0.9.

### 5.2. Results

RTs were left-skewed with a large *SD* (24.761 s). The mean RT was 7.248 s. The average median RT for each trial among all participants was 5.511 s (*SD* = 1.836 s). The 2 (trial type: switch vs. non-switch) × 2 (graph type: line vs. pie) × 2 (position difference of A and B: 1 vs. 2) ANOVA on median RTs per participant per condition showed a main effect of trial type, [*F*_(1, 59)_] = 6.39, *p* = 0.014, $\eta_{\text{p}}^{2}$ = 0.10, and an interaction of trial type × graph type, [*F*_(1, 59)_] = 5.21, *p* = 0.03, $\eta_{\text{p}}^{2}$ = 0.08, suggesting that non-switch trials were processed faster than switch trials when switching from line graphs to pie graphs (confirmed ***Hypothesis 1b***), and this effect was larger for pie graphs than for line graphs. There was a main effect of position difference, [*F*_(1, 59)_] = 6.57, *p* = 0.01, $\eta_{\text{p}}^{2}$ = 0.10, indicating quicker processing with adjacent groups than non-adjacent groups (confirmed ***Hypothesis 2b***). The interaction of trial type × position, [*F*_(1, 59)_] = 4.90, *p* = 0.03, $\eta_{\text{p}}^{2}$ = 0.08, reflected that this effect was larger in non-switch trials than in switch trials. No other effect was detected, graph type × position, [*F*_(1, 59)_] = 3.27, *p* = 0.08, $\eta_{\text{p}}^{2}$ = 0.05, graph type, and trial type × graph type × position, *Fs* < 1. The analysis of error rates did not show any main effect or interaction, indicating that line graphs had similar error rates as pie graphs (more details see Supplementary material).

**Figure 4:**
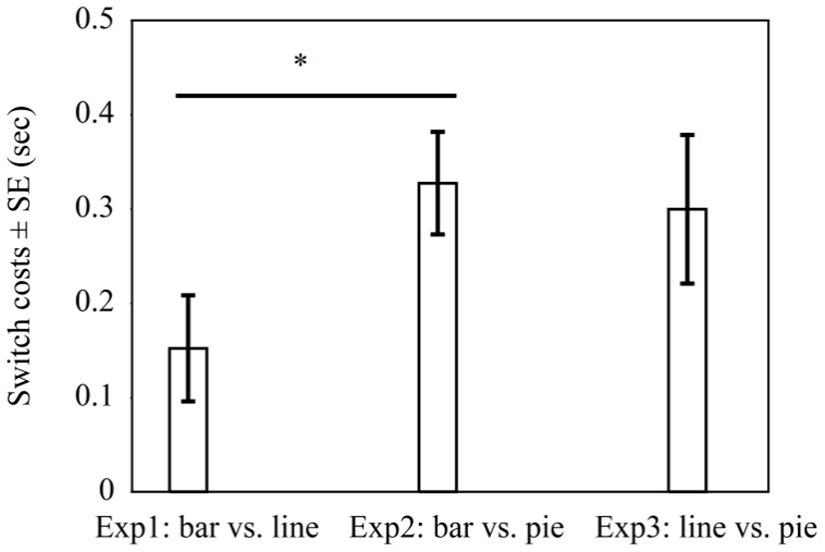
Average switch costs in seconds in Experiment 1 with bar graphs vs. line graphs, Experiment 2 with bar graphs vs. pie graphs, and Experiment 3 with line graphs vs. pie graphs. * indicates *p* < 0.05.

In all three experiments, switch costs were present. As this suggests that none of the graph types fully overlap in graph schema, we explored whether there might be differences in switch costs for the different pairings. This would be in line with a hierarchical view of graph schemas. The hierarchical view implies that graph schemas are organized hierarchically with a general schema and graph-specific schemas (cf. Pinker, 1990). Whenever switching from one graph type to another, the specific schema needs to change in any case. This could lead to at least *some* switch costs even when the general schema does not necessarily change. Conversely, the pairs where graphs differ in general *and* with respect to graph-specific schemas might show larger switch costs than the pairs that differ only in their specific graph schemas but share the same general graph schema. Accordingly, a post-hoc analysis was conducted to compare for differences in switch costs (RTs of non-switch trials subtracted from RTs of switch trials) across experiments. Figure 4 shows that the switch costs between bar graphs and line graphs in Experiment 1 (*M* = 0.152 s, *SD* = 0.435) were lower than the switch costs between bar graphs and pie graphs in Experiment 2 (*M* = 0.327 s, *SD* = 0.348), *t*_(99)_ = −2.15, *p* = 0.03. This result is consistent with the view that bar graphs and line graphs share the same general graph schema but differ in graph-specific schemas, while bar graphs and pie graphs might differ in general as well as with regard to specific schemas. Furthermore, the switch costs between line graphs and pie graphs in Experiment 3 (*M* = 0.300 s, *SD* = 601) did not differ significantly from the switch costs between bar graphs and line graphs in Experiment 1, *t*_(116)_ = −1.53, *p* = 0.13, and it did not differ from the switch costs between bar graphs and pie graphs in Experiment 2, *t*_(97)_ = 0.27, *p* = 0.79. Note however that the significant difference reported above does not prove robust when applying a Bonferroni-correction (see Discussion).

### 5.3. Discussion

Experiment 3 shows switch costs by pairing line graphs with pie graphs. Processing time was faster for pie graphs than for line graphs when comparing groups in a non-switch condition with a position difference of 1. Adjacent groups were compared quicker than non-adjacent groups. In addition, the comparisons of switch costs across experiments provided tentative evidence for a pattern consistent with the hierarchical account of graph schema (cf. Pinker, 1990). More specifically, switching between bar graphs and pie graphs seemed to lead to higher switch costs than switching between bar graphs and line graphs. Yet switching between line graphs and pie graphs did not differ in terms of the switch costs between bar graphs and line graphs, and between bar graphs and pie graphs. As the significant difference between Experiment 1 and 2 does not prove robust when applying a Bonferroni-correction, there is need for further empirical work. This should ideally address differences in switch costs in a fully randomized experimental design rather than in an across-experiment comparison.

## 6. General discussion

This study used a group comparison task involving the report of numerically exact group differences to test whether graph schemas are based on perceptual features or common invariant structures and to determine which graph type (bar, line or pie) is the most suitable for group comparisons. There are three main findings.

### 6.1. Graph schemas can be based on common invariant structures in a hierarchical structure

The study suggests that bar graphs and line graphs are more alike (in terms of the underlying schema) than pie graphs compared with bar or line graphs (i.e., pie graphs clearly fall under a different schema than bar and line graphs). A previous study by Ratwani and Trafton (2008) examined whether the assumptions that graph schemas are defined by a common invariant structure or by perceptual features by identifying the value of a group in bar, line, and pie graphs. In contrast, the present study used a discrete comparison task (cf. Zhao and Gaschler, 2021). Inconsistent with ***Hypothesis 1a***, bar graphs had no switch costs when paired with line graphs, but line graphs had switch costs when paired with bar graphs. Comparisons of switch costs across experiments revealed that the switch costs between bar graphs and line graphs were lower than the switch costs between bar graphs and pie graphs. Consistent with ***Hypothesis 1b***, pairing pie graphs either with bar graphs or line graphs led to switch costs, as pie graphs use a polar coordinate system. It is probable that there was a gradual effect with line graphs and bar graphs belonging to a more similar graph schema and pie graphs belonging to a distinct schema.

Moreover, this study provides tentative evidence supporting the hierarchical view of the graph schema (cf. Pinker, 1990), which suggests that graph schemas can be hierarchically structured with a general graph schema and graph-specific schemas. The general graph schema refers to common invariant features of all graphs, and the graph-specific schemas refer to the unique features of individual graphs. Figure 4 illustrates that pairing graphs that differ in general *and* graph-specific schemas (Experiment 2 bar vs. pie) led to greater switch costs than pairing graphs that differ mainly in graph-specific schemas (Experiment 1 bar vs. line). The hierarchical structure of graph comprehension also corresponds to studies on comprehension and memory, which demonstrate that human memory is organized in a hierarchical structure along a continuum from general to specific (Meyer, 1975; Carpenter and Just, 1977; Schank and Abelson, 1977; Kintsch and van Dijk, 1978). Our results suggest that bar graphs and line graphs are more alike (in terms of the underlying schema) than pie graphs compared with bar or line graphs (i.e., pie graphs clearly fall under a different schema than bar and line graphs).

### 6.2. Bar graphs are ideal for discrete comparison compared with line graphs and pie graphs

During discrete comparisons, participants are assumed to first scan the locations of relevant groups, then they project horizontal rays from the height of the bar or line graph to the axis from where they then extract the values of the relevant groups, and finally they compare the differences (Kosslyn, 1980; Ullman, 1984; Simkin and Hastie, 1987; Pinker, 1990; Gillan and Callahan, 2000). Consistent with ***Hypothesis 2a***, bar graphs allow for a quicker comparison of group differences than line graphs and pie graphs. The error analysis also suggested that bar graphs led to lower error rates than line graphs (in Experiment 1 bar vs. line). According to Cleveland and McGill (1984), bar graphs compared with other graphs (e.g., dot plots) allow for a better mapping of entities to distinct spatial locations. Bar graphs use individual entities to separate one group from other groups, and use heights to represent the values of each group (Pinker, 1990). Bar graphs are thus associated with discrete comparison (Zacks and Tversky, 1999). Line graphs use points to represent groups and heights to represent the values of each group, and the discrete data points are connected by lines (Pinker, 1990). Line graphs are thus associated with trends (Carswell et al., 1993; Zacks and Tversky, 1999). Accordingly, it is easier to pair discrete bars in bar graphs than compare the connected points in line graphs, as lines are interpreted as a single object rather than discrete data points based on the continuity Gestalt principle (Todorovic, 2008).

Pie charts use slices to represent groups and labels to represent values of groups (Gillan and Callahan, 2000). A pie chart uses area size to encode information. Viewers are more likely to spontaneously make proportional judgments with pie charts (Simkin and Hastie, 1987). As all pie slices have the same length of radii, viewers have to superimpose (i.e., mentally rotate until alignment, Spence and Lewandowsky, 1991) the angles of slices to compare the differences. In addition, labels in pies are displayed in a circular order, which is uncommon for referent extraction (Huestegge and Pötzsch, 2018). According to the principle of spatial alignment (Matlen et al., 2020), discrete comparison is more efficient when the required components in the graphs are in direct alignment. It is thus easier to compare heights of individual entities in bar graphs than slices in pie graphs.

Moreover, the task of discrete comparisons includes the time of graphical perception, which can be different for each graph (Kosslyn, 1980). For instance, perception of change is direct in bar graphs and line graphs, but not in pie graphs (Hollands and Spence, 1992). Horizontal pictographs (i.e., data points in rectangular form) are perceived more quickly and more accurately than vertical pictographs (Price et al., 2007). Nevertheless, one should be aware that although bar graphs are processed fast in discrete comparison tasks, they lead to biased judgement when estimating the means of all groups (Godau et al., 2016) and when judging if specific data points lie within a distribution (Newman and Scholl, 2012).

### 6.3. Adjacent groups are compared quicker than non-adjacent groups

Consistent with ***Hypothesis 2b***, position differences of groups of interests indeed affected processing time. Adjacent group comparisons required shorter processing time than non-adjacent group comparisons, which replicates the results of a previous study (Zhao and Gaschler, 2021). It also corresponds to the split-attention effect, which finds that larger saccades and more time are needed to integrate relevant information that is displayed separately (Chandler and Sweller, 1992; Mayer and Moreno, 1998; Johnson and Mayer, 2012). Several eye-tracking studies on graph comprehension also provided evidence on more transitions and longer fixations in integrative processes (i.e., infer quantitative relations) and suggested minimizing the overload of integrative processes. For instance, Carpenter and Shah (1998) measured the eye movements while participants interpreted and answered questions about line graphs. They suggested a sequential process of graph comprehension: pattern-recognition, interpretive, and integrative processes. During the integrative processes, participants showed considerable transitions on labels and values of the variables that determine relations. They thus recommended graphic designers reduce the effort required to identify the to-be-compared graphs. Huestegge and Philipp (2011) recorded eye movement patterns while participants judged the compatibility of data and statements in bar graphs and line graphs. Participants had fewer gaze transitions between data–legend compatible graphs than incompatible graphs, which suggests less difficulty in graph comprehension. Körner (2004, 2011) and Körner et al. (2014) conducted a series of eye–tracking studies on hierarchical graphs with nodes and relation lines (e.g., computer file systems, family trees). The results yielded that participants first search for relevant graph nodes and then solve problems by reasoning about the relationships (e.g., is a better than c?). They suggested supporting the serial cognitive processes of graph comprehension by decreasing the overload of search and integrative processes. Taken together, one implication is that we should, when possible, put the to-be-compared groups near each other to save processing time.

### 6.4. Limitations

This study has several limitations. We currently do not know which specific aspects of the graphs might have led to differences in switch costs. While bar and line graphs use a Cartesian coordinate system, a polar coordinate system is relevant in pie charts. This aspect might be the key difference among the graph schemas used for the different graph types. Yet, in order to support this assumption, one would need to include more comparisons of graphs with a polar and a Cartesian coordinate system. The graphs used in this study were taken from Ratwani and Trafton (2008) and have specific characteristics relevant for future work. Legends are displayed outside pie charts, values are labeled directly over the slices, and groups were displayed using different colors. The present results are potentially difficult to generalize to other forms of bar, line, and pie graphs (e.g., with and without direct labeling of values, labels; graphs with different specific arrangements or features, etc.). Further studies should examine the robustness of results by using bar graphs and line graphs with similar visual characteristics in terms of legends, labels and colors (cf. Shah and Hoeffner, 2002, for a review; Michal and Franconeri, 2017). Moreover, anchoring should be further examined by adding grid lines in bar and line graphs (cf. Schutz, 1961), as viewers tend to compare slices in pie charts by using 25%, 50%, or 75% anchors (Gillan and Callahan, 2000). The task in this study was to compare discrete groups based on one-variable data. It might be intriguing to consider multivariate data (e.g., three-variable data), as viewers tend to give different descriptions regarding main effects and interactions when viewing bar and line graphs (Shah and Freedman, 2009). The positions of depicted groups were randomized in this study, which makes it difficult to identify a particular trend evolving from group A to group B. Future studies should use more data points with different numbers of trend reversals (i.e., slopes of adjacent lines from positive to negative or vice-versa), as it was shown that they have an impact on comprehension time (Carswell et al., 1993). Future studies should also examine how schema switches might affect graph processing when a single task involves comparisons between multiple (similar or different) graphs, that is, in complex graph display (e.g., see Riechelmann and Huestegge, 2018; Poetzsch et al., 2020). Other types of tasks should be used in future studies, such as a more basic “which is larger” comparison, A + B vs. C + D, as pie charts are ideal to combine even non-adjacent slices compared to summing up heights in bar graphs (Spence and Lewandowsky, 1991). Further studies should compare vertical and horizontal bars, as previous studies showed that horizontal bars are slightly preferred to and less biased than vertical bars (Culbertson and Powers, 1959; Cai et al., 2017). Lastly, the age, education and work experience of the subjects should be considered in the future.

### 6.5. Conclusion

Using graphs (e.g., bars, lines, or pies) to compare quantitative data is common, especially in the media. This study suggests that bar graphs and line graphs are more alike (in terms of the underlying schema) than pie graphs compared with bar or line graphs (i.e., pie graphs clearly fall under a different schema than that of bar and line graphs). Moreover, this study shows tentative evidence for the hierarchical structure of graph schemas. Bar graphs are more effective than line graphs and pie graphs in discrete comparisons due to the specific graphical patterns of individual entities and values represented by heights. In addition, this study provides the implication to place to-be-compared groups adjacently to save processing time. This can be especially important for contexts where speed and accuracy are highly relevant, such as when estimating the survival odds for a treatment (Price et al., 2007).

## Data availability statement

The datasets for this study can be found in the Open Science Framework: https://osf.io/nsj32.

## Ethics statement

The studies involving human participants were reviewed and approved by the Ethics Review Board of the Faculty of Psychology at the FernUniversität (August 8, 2019). The patients/participants provided their written informed consent to participate in this study.

## Author contributions

FZ has conceptualized the study, written the program, organized the data collection, analyzed the data, and prepared the paper. FZ and RG have jointly edited the manuscript and approved its submission.

## Footnotes

^1^The analyses including three trial types (pure vs. switch vs. non-switch) in 3 Experiments can be found in the Appendix. Note that the order of the blocks was not counterbalanced in the study and pure blocks occurred most often (90% of the time in Experiment 1; 95.1% in Experiment 2; 89.7% in Experiment 3) in the first and second blocks. Potentially as a result of general practice effects, RTs were much longer in pure blocks than mixed blocks in Experiment 1 (pure: 4.904 s ± 2.578; mixed: 4.521 s ± 1.178, *t*(59) = 3.95, *p* < .001, two-tailed), and in Experiment 2 (pure: 5.600 s ± 1.679; mixed: 5.773 s ± 2.050, *t*(40) = 3.25, *p* =.002). Pure blocks and mixed blocks did not differ in Experiment 3 (pure: 5.773 s ± 2.050; mixed: 5.569 s ± 2.034, *t*(57) = 1.54, *p* =.13). This might reflect a general practice effect (early blocks slower than later blocks) rather than mixing cost.
