# Supplementary material for: Graph schema and best graph type to compare discrete groups: Bar, line, and pie
Source: Front Psychol. 2022 Dec 19;13:991420. doi: 10.3389/fpsyg.2022.991420 (PMC9806344; doi:10.3389/fpsyg.2022.991420)
Supplement: Supplementary file 1 [file Data_Sheet_1.docx]

Supplementary Material

# Analyses of error rates

**Table 1A**

Average error rates (in %) between difference of positions of group A and group B (DiffPos) in pure trials and switch and nonswitch trials for Experiments 1-3.

|  | Pure  Block | | Mixed Block | | | |
| --- | --- | --- | --- | --- | --- | --- |
|  |  |  | Switch | | Nonswitch | |
| Graph Type | DiffPos1 | DiffPos2 | DiffPos1 | DiffPos2 | DiffPos1 | DiffPos2 |
| **Exp. 1 (*N* = 60)** |  |  |  |  |  |  |
| Bar *M* | 5.4% | 3.4% | 2.3% | 0.6% | 2.7% | 1.3% |
| *(SD)* | (4.0%) | (4.4%) | (5.5%) | (4.3%) | (5.3%) | (5.5%) |
| Line | 8.7% | 8.7% | 7.4% | 6.8% | 5.2% | 6.0% |
|  | (6.6%) | (8.4%) | (11.9%) | (12.1%) | (6.1%) | (9.9%) |
| **Exp. 2 (*N* = 41)** |  |  |  |  |  |  |
| Bar | 8.2% | 4.8% | 5.5% | 2.8% | 4.2% | 2.4% |
|  | (5.1%) | (6.6%) | (8.4%) | (9.6%) | (7.9%) | (7.5%) |
| Pie | 7.3% | 8.0% | 4.2% | 3.4% | 4.2% | 2.0% |
|  | (8.3%) | (17.6%) | (8.6%) | (8.8%) | (4.7%) | (6.0%) |
| **Exp. 3 (*N* = 58)** |  |  |  |  |  |  |
| Line | 10.1% | 10.3% | 7.1% | 6.9% | 6.8% | 7.9% |
|  | (14.2%) | (15.8%) | (17.7%) | (19.8%) | (15.7%) | (20.0%) |
| Pie | 8.8% | 8.9% | 8.5% | 6.9% | 6.3% | 6.1% |
|  | (14.8%) | (16.0%) | (12.7%) | (15.6%) | (11.8%) | (13.3%) |

## Excluding pure blocks

**Experiment 1: Bar vs. Line.** The average error rate for each trial among all participants was 4.0% (*SD* = 3.5%). The 2 (trial type: switch vs. nonswitch) × 2 (graph type: bar vs. line) × 2 (position difference of A and B: 1 vs. 2) ANOVA on average error rates per participant per condition showed only a main effect of graph type, *F*(1, 59) = 25.49, *p* < .001, η_p_^2^ = .30, suggesting bar graphs had lower error rates than line graphs in group comparison. No other effect was found, trial type, trial type × position, graph type × trial type × position, *Fs* <1, position, *F*(1, 59) = 1.04, *p* = .31, η_p_^2^ = .02; trial type × graph type, *F*(1, 59) = 3.55, *p* = .07, η_p_^2^ = .06; graph type × position, *F*(1, 59) = 1.77, *p* = .19, η_p_^2^ = .03.

**Experiment 2: Bar vs. Pie.** The average error rate for each trial among all participants was 3.6% (*SD* = 3.4%). The corresponding ANOVA on error rates revealed only a main effect of position, *F*(1, 40) = 5.05, *p* = .03, η_p_^2^ = .11, suggesting larger position difference leads to lower error rates. No other effect was found, trial type, graph type, trial type × graph type, trial type × position, graph type × position, graph type × trial type × position, *Fs* < 1.

**Experiment 3: Line vs. Pie.** The average error rate for each trial among all participants was 7.1% (*SD* = 11.3%). The corresponding ANOVA on error rates did not reveal any main effect or interaction. No other effect was found, trial type, graph type, position, trial type × position, graph type × position, graph type × trial type × position, *Fs* < 1, trial type × graph type, *F*(1, 57) = 1.69, *p* = .20, η_p_^2^ = .03.

## Including pure blocks

**Experiment 1: Bar vs. Line.** The average error rate for each trial among all participants was 5.9% (*SD* = 3.2%). The 3 (trial type: pure vs. switch vs. nonswitch) × 2 (graph type: bar vs. line) × 2 (position difference of A and B: 1 vs. 2) (see Table 1 and Figure 3) ANOVA on average error rates per participant per condition showed the main effect of trial type, *F*(2, 118) = 9.66, *p* < .001, η_p_^2^ = .14, indicating the higher error rates in pure trials than in switch and nonswitch trials (see Table 1A). There was a significant effect of graph type, *F*(1, 59) = 42.36, *p* < .001, η_p_^2^ = .42, suggesting bar graphs had lower error rates than line graphs in group comparison. No other effect was found, position, *F*(1, 59) = 1.97, *p* = .17, η_p_^2^ = .03; trial type × graph type, *F*(1.77, 104.14) = 1.47, *p* = .24, η_p_^2^ = .02; trial type × position, *F* < 1; graph type × position, *F*(1, 59) = 3.44, *p* = .07, η_p_^2^ = .06; graph type × trial type × position, *F* < 1.

**Experiment 2: Bar vs. Pie.** The average error rate for each trial among all participants was 6.2% (*SD* = 4.9 %). The corresponding ANOVA on error rates revealed a main effect of trial type, *F*(1.74, 69.46) = 6.94, *p* = .003, η_p_^2^ = .15, indicating the lowest error rates in nonswitch trials than in pure and switch trials. There was a significant effect of position, *F*(1, 40) = 5.62, *p* = .02, η_p_^2^ = .12,suggesting larger position difference leads to lower error rates. No other effect was found, graph type, trial type × graph type, trial type × position, graph type × trial type × position, *Fs* < 1, graph type × position, *F*(1, 40) = 2.85, *p* = .10, η_p_^2^ = .07.

**Experiment 3: Line vs. Pie.** The average error rate for each trial among all participants was 7.9% (*SD* = 11.6 %). The corresponding ANOVA on error rates revealed a main effect of trial type, *F*(2, 114) = 5.85, *p* = .004, η_p_^2^ = .009, indicating the highest error rates in pure trials than in nonswitch and switch trials. No other effect was found, graph type, position, trial type × graph type, trial type × position, graph type × position, graph type × trial type × position, *Fs* < 1.

# Analyses of reaction time including pure blocks

**Experiment 1: Bar vs. Line.** A repeated-measures ANOVA was conducted on median RTs per participant per condition with the following factors: 3 (trial type: pure vs. switch vs. nonswitch) × 2 (graph type: bar vs. line) × 2 (position difference of A and B: 1 vs. 2) (see Table 1 and Figure 3). Trial type reached significance, *F*(1.35, 79.59) = 13.67, *p* < .001, η_p_^2^ = .19 [here and elsewhere the greenhouse-Geisser correction was applied], and the interaction of trial type × graph type, *F*(1.66, 98.05) = 22.58, *p* < .001, η_p_^2^ = .28. It suggested trial type affected the processing times on bar graphs and line graphs differently. While bar graphs were processed at similar speed across the three trial types, line graphs were processed quicker with nonswitch trials than pure and switch trials. Furthermore, there was a significant effect of graph type, *F*(1, 59) = 146.15, *p* < .001, η_p_^2^ = .71, indicating shorter processing time in bars to compare groups than in lines. The effect of position difference was significant, *F*(1, 59) = 45.19, *p* < .001, η_p_^2^ = .43, as well as the interactions of trial type × position, *F*(2, 118) = 3.15, *p* = .047, η_p_^2^ = .05, and trial type × graph type × position, *F*(1.71, 100.69) = 4.29, *p* = .02, η_p_^2^ = .07, indicating longer RTs with the increase of position difference and the different effect of position on graph type and trial type. No other effect was found, graph type × position, *F*(1, 59) = 1.80, *p* = .19, η_p_^2^ = .03.

**Experiment 2: Bar vs. Pie.** The 3 (trial type: pure vs. switch vs. nonswitch) × 2 (graph type: bar vs. pie) × 2 (position difference of A and B: 1 vs. 2) ANOVA on median RTs per participant per condition yielded the main effect of trial type, *F*(1.70, 67.91) = 9.11, *p* = .001, η_p_^2^ = .19, suggesting that the processing time was the quickest in non-switch trials than switch and pure trials. There was a significant effect of graph type, *F*(1, 40) = 26.51, *p* < .001, η_p_^2^ = .40, indicating shorter processing time in bars to compare groups than in pies. The effect of position difference was significant, *F*(1, 40) = 14.94, *p* < .001, η_p_^2^ = .27, as well as the interactions of trial type × position, *F*(2, 80) = 5.07, *p* = .01, η_p_^2^ = .11, indicating longer RTs with the increase of position difference and the larger effect of position on nonswitch trials than on pure and switch trials. No other effect was found, graph type × position, *F*(1, 40) = 2.23, *p* = .14, η_p_^2^ = .05, trial type × graph type, and trial type × graph type × position, *Fs* < 1.

**Experiment 3: Line vs. Pie.** The 3 (trial type: pure vs. switch vs. nonswitch) × 2 (graph type: line vs. pie) × 2 (position difference of A and B: 1 vs. 2) ANOVA on median RTs per participant per condition showed the main effect of trial type, *F*(1.68, 96.01) = 3.71, *p* = .04, η_p_^2^ = .06, suggesting that the processing time was quicker in non-switch trials than pure and switch trials. The effect of position difference was significant, *F*(1, 57) = 18.60, *p* < .001, η_p_^2^ = .25, as well as the interactions of trial type × position, *F*(1.75, 99.54) = 3.68, *p* = .03, η_p_^2^ = .06, indicating longer RTs with the increase of position difference and the larger effect of position on nonswitch and pure trials than on switch trials. No other effect was found, graph type, *F*(1, 57) = 2.15, *p* = .15, η_p_^2^ = .04, trial type × graph type, *F* < 1, graph type × position, *F*(1, 57) = 3.22, *p* = .08, η_p_^2^ = .05, trial type × graph type × position, *F*(1.81,102.91) = 1.18, *p* = .31, η_p_^2^ = .02.
